# Patient Perspective on Use of an Interactive Website for Sleep Apnea

**DOI:** 10.1155/2013/239382

**Published:** 2013-03-14

**Authors:** Carl Stepnowsky, Christine Edwards, Tania Zamora, Robert Barker, Zia Agha

**Affiliations:** ^1^Health Services Research and Development Unit, Veteran Affairs San Diego Healthcare System, 3350 La Jolla Village Drive 111N-1, San Diego, CA 92161, USA; ^2^Department of Medicine, University of California, San Diego, CA 92037, USA

## Abstract

Incomplete patient adherence with nasal continuous positive airway pressure (CPAP) limits the effectiveness of treatment and results in suboptimal obstructive sleep apnea (OSA) outcomes. An interactive website specifically designed for patients with OSA was designed and utilized in a randomized clinical trial to test its effect on increasing CPAP adherence. The goal of this paper is to report on CPAP adherence, internet use, privacy concerns and user satisfaction in using the website. The original project was designed as a randomized, controlled clinical trial of Usual Care (UC, control) versus MyCPAP group (intervention). Questionnaires were administered to evaluate the patient perspective of using the MyCPAP website. Participation in the MyCPAP intervention resulted in higher CPAP adherence at the two-month time point relative to participation in the UC group (3.4  ±  2.4 and 4.1  ±  2.3 hrs/nt; *P* = 0.02; mean  ±  SD). Participants randomized to the MyCPAP website increased their use of the internet to obtain OSA related information, but did not increase their use of the internet to get information on general health or medical conditions. Users had very little concern about their CPAP data being viewed daily or being sent over the internet. Future studies should consider the use of newer evaluation criteria for collaborative adaptive interactive technologies.

## 1. Introduction

Obstructive sleep apnea (OSA) is a disorder characterized by repeated cessations of breathing during sleep, which can result in a number of potentially serious consequences affecting cardiovascular, physiological, neurocognitive, emotional, and psychosocial functioning [1]. OSA is the most common sleep disorder, affecting about 4% of men and 2% of women aged from 30 to 70 years old in the USA [2]. OSA is a chronic disease that is estimated to contribute $3 billion in additional medical costs in the USA, with a total economic burden greater than $100 billion when including loss of workplace productivity, occupational injury, and greater health care utilization [3]. In addition to its economic burden, OSA is associated with serious long-term adverse health consequences such as hypertension [4], metabolic dysfunction [5], cardiovascular disease [6], neurocognitive deficits [7], and motor vehicle accidents [8]. 

Nasal continuous positive airway pressure (CPAP) [9] is the treatment of choice for OSA [10], with meta-analytic reports of numerous randomized controlled trials showing that CPAP improves both objectively and subjectively measured daytime sleepiness [11] as well as health-related quality of life [12]. CPAP has been shown to normalize sleep architecture [13] and reduce blood pressure [14]. Emerging evidence suggests that CPAP treatment reduces physician's costs and hospital utilization rates in the two years after the start of treatment in OSA patients when compared to age-, gender-, and location-matched controls [15]. 

Despite the documented efficacy of CPAP, many—perhaps most—patients have difficulty adhering. It is estimated that over 50% of those started on CPAP will not be using it one year later [10]. A number of CPAP adherence interventions have been studied to date, with most offering extra education or clinical support [16]. Some of these are considered intensive or augmented support [17, 18] and require significant extra time by the provider, upwards of 20 hours of extra contact or more. These kinds of interventions are difficult to incorporate into our fee-for-service healthcare system. We took the opportunity to design an interactive website to help automate educational and clinical support efforts and add the ability to track CPAP adherence data and OSA-related symptoms. 

Identifying the factors or correlates associated with increased CPAP adherence could help inform more effective interventions. Most studies examining the correlates of CPAP adherence have considered patient-related, disease-related, and treatment-related variables [16, 19, 20]. No reliable (i.e., consistent) and modifiable (i.e., amenable to change) determinants measured *prior* to treatment initialization have been found in any of these categories [19, 20]. Interestingly, while the main complaints from patients concern comfort related issues (i.e., mask fit, pressure tolerance, dry mouth), any technological solution (i.e., improvements in mask design, autoadjusting pressure or pressure relief, and humidification) all have been found to have little to no impact on CPAP adherence and outcomes [21]. Use of autoadjusting PAP has been found to improve adherence on average 11 minutes per night, which is not a clinically important amount of use [21]. However, there is increasing evidence that variables measured after the start of treatment might predict future adherence. For example, the amount of perceived benefit from treatment (e.g., improvement in sleepiness level) is associated with higher CPAP adherence [22–24]. A past review of the literature pointed to the need for increased research on the role of psychosocial variables in CPAP adherence behavior [20]. Research examining the relationship between social-cognitive variables is accumulating. Social cognitive variables are associated with CPAP adherence in studies of first-time CPAP users when CPAP adherence measured both at one month [25] and at 3 and 6 months [26]. Another study found that cognitive beliefs (health value, health locus of control, self-efficacy) are associated with CPAP adherence at 3 months in new users [27]. 

The advantage to this class of CPAP adherence factors are that they are theoretically-based, modifiable, and can provide the basis for interventions to improve use of CPAP [28]. Our group has begun work on a Sleep Apnea Self-Management Program that is based on this previous work [28]. The intervention described in this manuscript is based in part on lessons learned from the Sleep Apnea Self-Management Program and is in part adapted to an interactive website format. 

The percentage of adults in the United States that use the Internet was approximately 74% in 2011, with no difference between men and women [29]. Of those American adults who use the Internet, 80% look for health or medical information such as a specific disease or treatment [30]. This translates to 59% of all US adults. Information sought includes that regarding a specific disease or medical problem (66%) or a certain medical treatment or procedure (56%). Increasingly, online users are becoming more active users (i.e., doing more than just reading): 14% have signed up to receive email updates, 6% have posted comments, and 25% are now accessing videos to learn more about health and medical issues. Two factors associated with higher rates of participatory activities online include: (a) internet users with 3 or more chronic conditions and (b) those who access the internet wirelessly. In an earlier study those with chronic conditions are more likely than other patients to report that their online searches affected their treatment decisions (75% versus 55%), their interactions with their doctors (69% versus 52%), and their ability to cope with their condition (57% versus 36%), as well as their diet and physical activity regimen (56% versus 42%). 

There are less positive data to report. While patients with chronic conditions do have positive experiences with their online health searches, 30% said they felt overwhelmed by the amount of information they found online and 31% said they felt frustrated by the lack of information or their inability to find what they were looking for [31]. Further, 80% said the most common barrier to health/medical websites are concerns about the information quality [32]. These issues speak to the need for websites to better understand what information users are looking for online, the need to organize and display that information in a user-friendly manner, and the need to address issues concerning the accuracy and quality of the information presented.

The goal of the manuscript was to describe the use of the MyCPAP website for OSA patient management and the effect on CPAP adherence and outcomes.

## 2. Methods

### 2.1. Overview

The design was a randomized parallel group trial with blinded evaluation that compared an Internet intervention based on the wireless telemonitoring of CPAP data (i.e., Internet-based positive airway pressure care, or MyCPAP) versus a usual care CPAP treatment protocol (i.e., Usual Care, or UC). Participants completed a baseline, 2-month and 4-month assessments. The project took place over a 3-year period. Usual care was comprised of pre-determined clinical contacts while MyCPAP was comprised of as-needed clinical contacts, based on objectively measured CPAP adherence and efficacy data and access to a patient-oriented Web site. Participants underwent identical instruction and education on OSA and CPAP therapy and used identical CPAP units. The study was designed as a practical clinical trial that compared one clinical care method against another, with the goal of informing clinical decision making [33]. It was comparing the effect of clinical care methods on a behavioral outcome (i.e., CPAP adherence) and was considered in large part a behavioral trial.

### 2.2. Participants

The target population for this study was all patients referred to the University of California, San Diego Healthcare System (UCSD) Sleep Medicine Center, by physicians for suspicion of OSA. Participants were recruited and screened in the Sleep Center. Inclusion criteria included a diagnosis of OSA (apnea-hypopnea index ≥ 15) [34], CPAP therapy prescription, and age ≥ 18 years. Exclusion criteria included residence in a geographical area outside of San Diego County (which could make regular contact and participation difficult); fatal comorbidity (life expectancy less than 6 months as indicated by treating physician); or significant documented substance/chemical abuse. All participants signed informed consent and the study was approved by the University of California, San Diego IRB. The participants were offered financial reimbursement for participating and completing the study and to modestly offset travel-related expenses.

### 2.3. Intervention

Participants randomized to UC were followed according to both the usual and standard care for OSA patients who are treated by the UCSD Sleep Center and by the published literature [10]. These standards include diagnostic sleep study, CPAP instruction and setup by trained health care provider, and followup at predetermined times (1 week, 1 month) by CPAP clinic staff. Beyond these pre-determined clinic contacts, patients were encouraged to call whenever they had a problem or concern. Adjustments or changes in the mask interface might be warranted at any point, so it is not uncommon for patients to switch from nasal to full-face masks or nasal pillows, for example. Pressure level changes are often warranted as well. If the patient brought in their CPAP unit, the data was downloaded and utilized.

An individual working for a VASDHS-contracted home medical supply company conducted comprehensive CPAP instruction in a group format per the study protocol. Participants were given a choice between full face and nasal mask types, given the opportunity to wear the masks, experience the positive airway pressure, and work through any initial problems or issues. Each CPAP used in this study was equipped with a digital data smart card, which recorded the amount of time the machine was used therapeutically. Data were downloaded from the smart card after the four-week intervention period to measure the amount of daily CPAP use.

### 2.4. MyCPAP Website

The main goals of the MyCPAP intervention was to (a) allow both the patient and provider access to telemonitored adherence and efficacy data on a daily basis, (b) act on that data collaboratively to guide CPAP management and troubleshoot problems early and effectively, and (c) emphasize ways for the patient to express their preferences and needs. Below we describe both the patient and provider portals, which are set up differently given the different needs of patients and providers.  Table 1 provides a list of the MyCPAP components.

Patients randomized to MyCPAP had objective CPAP data monitored as frequently as every day throughout the active 2-month treatment period. The frequency and nature of the clinical interactions were largely dependent upon patient-defined needs, subjective patient report of symptoms and progress, and the objectively measured nightly data values. Participants were assigned a unique username and password to access the website and provided with an initial overview of the website and its components. They were told that they could access the learning center to learn more about sleep apnea and CPAP, track their CPAP adherence and efficacy data, track symptoms that were important to them, troubleshoot problems that they experienced in using CPAP and access an animated site to learn how to use their device and mask. They were not required to use the site and there was no penalty nor incentive to use the site. 

### 2.5. Apparatus

Participants in this study were provided with a Positive Airway Pressure device (PAP; Autoset II, ResMed, San Diego, CA). A wireless modem was attached to their PAP device, which could then send the data from the device to a Web-portal accessible by our team. The web-portal (“Restraxx Data Center,” or RDC), is comprised of the wireless module and the server/database, which houses the data and, fully compliant with the Health Insurance Portability and Accountability Act of 1996 (HIPAA) restricts access to authorized health care professionals. The wireless module connects to the flow generator via a docking mechanism that allows the connection to an existing 15-pin expansion port at the rear of the flow generator. All PAP devices were outfitted with a humidifier.

### 2.6. Measures

Measures were assessed at both pre- and postintervention and included participant sociodemographics, OSA symptoms, Epworth Sleepiness Scale (ESS), Sleep Apnea Quality of Life Index (SAQLI) and the Center for Epidemiologic Studies—Depression (CES-D), computer use and experience survey, and items that assessed patient satisfaction with MyCPAP. Demographic information assessed included age, gender, education, marital status, height, and weight. The apnea-hypopnea index (AHI) is a count of the total number of apneas and hypopneas per hour of sleep and was measured by overnight sleep study.

The Epworth Sleepiness Scale (ESS) is an 8-item validated measure of daytime sleepiness [35]. It asks respondents to estimate how likely they are to doze in 8 different situations. The ESS is able to discriminate the sleepiness level of OSA patients from that of normal [35]. The score is based on a 0–24 point scale, with higher scores representing greater levels of sleepiness. Self-rated sleepiness was also assessed via a modified Visual Analog Scale (VAS). The score was the number circled on a range from 0 to 10, with the higher score indicating more sleepiness.

#### 2.6.1. Sleep Apnea Quality of Life Index (SAQLI)

 The SAQLI is an OSA-specific measure of health-related quality of life that is comprised of several sections: (1) Domain A: 14-item measure of daily activities, social interactions, and emotional functioning; (2) Domain B: OSA symptom list; and (3) Domain C: treatment-related symptom list. At baseline, the SAQLI total score is comprised of domains A and B [36, 37]. Once on therapy, domain C is assessed and included in the total score. 

Depressive symptoms were measured using the Center for Epidemiological Studies-Depression Scale short form (CES-D). The CES-D is a 10-item self-report measure of depression [38]. The 10-item version has adequate predictive accuracy when compared to the original full-length 20-item version, as well as adequate test-retest correlations and discriminative validity [39]. 

#### 2.6.2. Computer Use and Experience Survey

The Computer Use and Experience Survey was given to participants at baseline and 4 month followup. This 14-item questionnaire is designed to assess participants' familiarity with using a computer and the internet. Participants are also asked whether they use the internet to search for health-related information and whether they feel confident in the information that they find [40]. 

#### 2.6.3. Patient Satisfaction

This short questionnaire was administered at the 4-month followup to assess satisfaction with various aspects of the intervention, including the technical and personal manner of the provider with whom they interacted, the likelihood of continuing to use CPAP and whether they had concerns about their CPAP data being viewed daily be research staff or whether they had concerns about their data being sent over the internet [41]. 

#### 2.6.4. MyCPAP OSA Symptom Tracking

Participants could track up to 5 of their most important OSA-related symptoms on the MyCPAP website. OSA symptoms were originally selected from a list of 25 symptoms from the symptom list of the Sleep Apnea Quality of Life Index [37]. The most important 5 symptoms where then rated in terms of how much of a problem each symptom has been for the patient, and were anchored by the following rating scale: 1 = no problem and 5 = severe problem. Participants were given the option to track these symptoms on a weekly basis.

## 3. Results

Two hundred forty-one participants were enrolled over the project period (115 to Usual Care and 126 to the MyCPAP group). The total number of withdrawals during the course of the project was seven. These were due to CPAP intolerance or subsequent self-withdrawal from the study. Baseline rates of OSA patients with CPAP intolerance or refusal is estimated to be about 20% in clinical practice. In our project, this worked out to be about 3%, so appeared to be significantly lower. 

The average Body Mass Index (BMI) was 32.5 indicating that the majority of the participants enrolled in the study were overweight (defined as having a BMI greater than 30). The average Apnea-Hypopnea Index (AHI) for participants in the study was 36.5, which is categorized as being within the severe level of sleep apnea. The mean ESS score was 10.6, indicating that most participants had a significant level of excessive daytime sleepiness. There were no significant differences at baseline between the groups on age, BMI, AHI, or ESS (see Table 2).

### 3.1. CPAP Adherence

**Figure 1:**
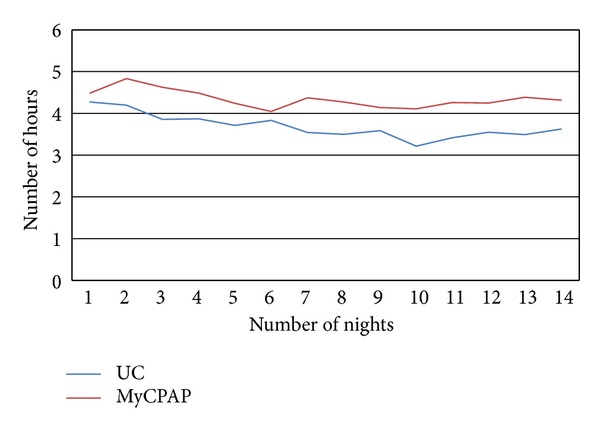
Adherence data over first 14 nights of CPAP usage.

**Figure 2:**
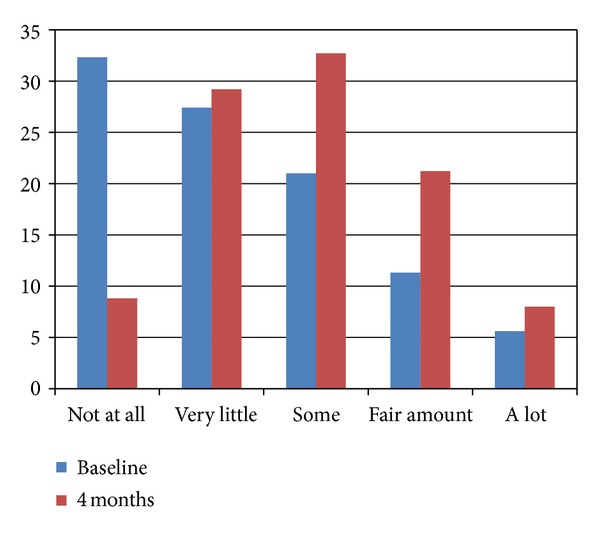
Frequency of response to how often the internet is used to get information on sleep apnea, at both baseline and four months (intervention group, only).

Figures 1 and 2 show the difference in CPAP adherence between the two groups at the 2-month and 4-month time points. Those who were randomized to the MyCPAP used CPAP on average nearly one hour per night longer than those in the Usual Care group at the 2-month time point (3.4 ± 2.4 and 4.1 ± 2.3 hrs/nt; *P* = 0.02; mean ± SD)., and those results held at the 4-month time point (3.2 ± 2.4 versus 3.9 ± 2.3 hrs/nt; *P* = 0.03). 

Figure 1 provides the nightly data from the first 14 days of use. Those randomized to the MyCPAP intervention appear to have a slight increase in use early in the treatment initialization process and that effect appears to sustain itself over time. These data are important because the literature suggests that adherence patterns are established relatively early in the treatment initialization process.

Despite the difference in adherence in the two groups, no differences were found on self-reported measures of OSA symptoms.  Table 3 provide data on the Epworth Sleepiness Scale, Sleep Apnea Quality of Life Scale, and the Center for Epidemiological Studies—Depression.

### 3.2. MyCPAP Group and Internet Use

Figure 2 provides the frequency of response to how often the internet was used to get information on sleep apnea at baseline and at the 4-month time point. The data shows that at baseline approximately 38% searched the internet for OSA information (21% “some,” 11% “fair amount,” and 6% “a lot”). At 4 months, the percentage who reported using the internet to search for OSA information increased to 62% (33% “some,” 21% “fair amount,” and 8% “a lot”). One can see the shift in the distribution from baseline to the 4-month time point.

**Figure 3:**
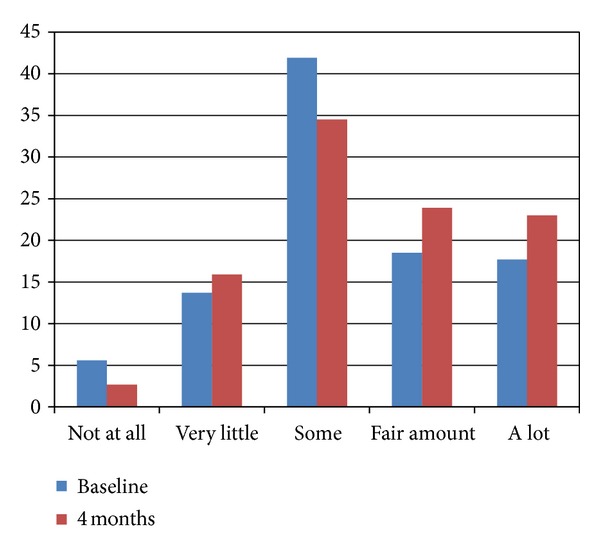
Frequency of response of how often the internet is used to get information on health, at both baseline and at four months (intervention group, only).

Figure 3 provides the frequency of response to how often the internet was used to get information on health in general at baseline and at the 4-month time point. The data shows that there was very little difference in the frequency with which the internet was used to access general information at health between baseline and the 4-month time point.

**Figure 4:**
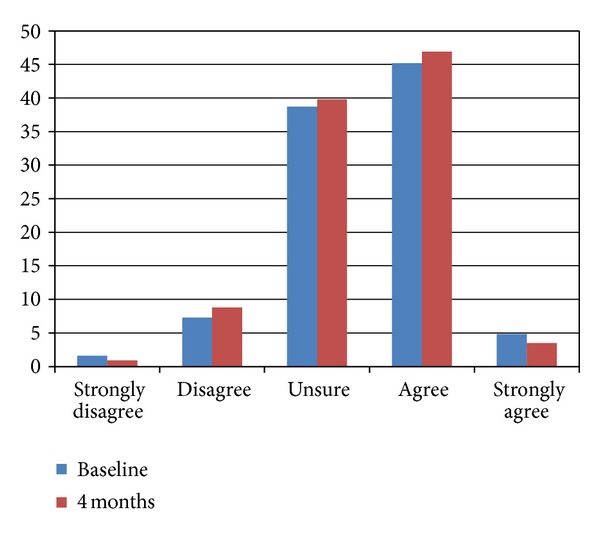
Frequency of response of the confidence about the accuracy of the health information found on the internet at baseline and at four months.

Figure 4 provides the frequency of response to the confidence of the participants about the accuracy of the health information found on the internet and how often the internet was used to get information on health in general at baseline and at the 4-month time point. The data shows that the there was very little change in the confidence of the accuracy for general health information on the internet across the time points, with approximately 50% agreeing or strongly agreeing at each time point. About 40% said they were not sure about the accuracy of the health information on the internet.

### 3.3. OSA Symptom Tracking

**Figure 5:**
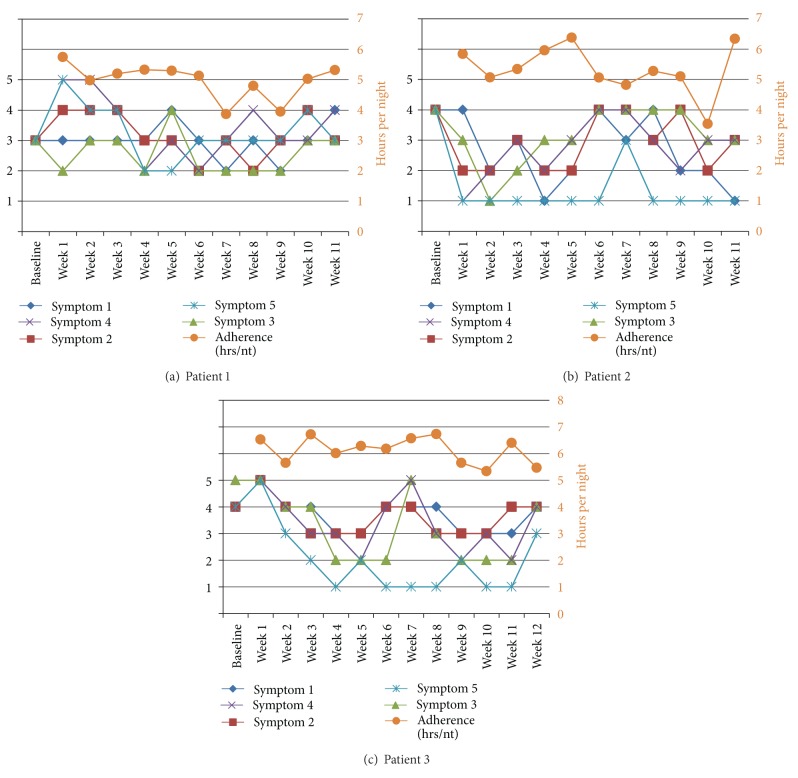
Tracking sleep apnea symptoms over time: example of three users.

One aspect of the MyCPAP website was the ability for the participants to track their OSA symptoms over time. Users could select the symptoms that most affected them, and then track those symptoms over time on a rating scale that was anchored with 5 being a severe problem and 1 being no problem. Figures 5(a)–5(c) includes baseline ratings of those symptoms. The figures are line graphs of both the tracking of the symptoms over time (1–5 scale) and CPAP adherence (in hrs/nt). The data from these three case examples were included because each included over 11 weeks of symptom tracking and CPAP adherence of greater than 4 hours per night. While each of the three cases show some symptom improvement, even in good users of CPAP, only one symptom is rated as being very low in its impact. These data provide support for the idea that CPAP, even in those with adequate or better CPAP adherence, may help to control OSA during the time it is used, but may ultimately ineffectively manage all sleep-related problems that an OSA patient may be experiencing. This data are important for the sleep physician to monitor and work collaboratively with the patient to find ways to improve OSA clinical management. 

### 3.4. Patient Satisfaction and Privacy

Several items assessed the satisfaction of the participants in the MyCPAP group on the technical skills and personal manner of the provider responsible for the care given in this group. For technical skills (thoroughness, carefulness, competence), only 5.6% rated these skills as fair or poor, and 94.4% rated them as good or better. For the personal manner (i.e., courtesy, respect, friendliness), 2.8% rated these skills as poor, 9.3% as fair, and 88% rated them as good or better. In terms of likelihood of continuing to use CPAP in the future, 64% rated themselves as “extremely” likely to continue to use CPAP and 21% rated themselves as “highly” likely. Because the MyCPAP website required use of the internet to track both CPAP data and their own symptom data, patients were asked about their concerns about viewing the data daily or about being concerned about the information being sent over the internet. The majority of users indicated that they were “not at all” concerned about these issues. 

## 4. Discussion

The study examined the patient perspective in using the MyCPAP website to help manage OSA. The study found that those sleep apnea patients who were randomized to the MyCPAP group had higher levels of CPAP use than those in the Usual Care group. When the nightly data over the first 2 weeks was plotted, it appeared that the use of the website had an effect that began early in the treatment initialization process and was sustained over time. 

Despite the significant change in CPAP adherence, there were no differences on the self-reported measures of OSA symptoms. The two groups did not differ at baseline on any of these measures, which meant that randomization worked in that the groups were similar on sleepiness level, OSA-specific quality of life and on depressive symptoms. It may be that a difference of one hour of CPAP use has minimal effect on improving OSA symptoms. Alternatively, it may be that the usual care group's level of CPAP use is enough to improve OSA symptoms. Rather than look at group differences, if we look at the magnitude of change for the groups, we see that for the Epworth Sleepiness Scale, both groups dropped about three points, to a mean of about 8. The cut-off for ESS is about 10, so that anything below is considered to be low sleepiness. For the SAQLI, the SAQLI user manual suggests that a change of 0.1 is a clinically meaningful change. If this is indeed the case, then both groups improved a clinically meaningful amount (each improved by 0.2 points on the SAQLI total score).

Future studies will need to evaluate the change in symptoms over longer periods of time to see whether the use of an intervention has a longer-term impact on OSA symptoms. One might expect that if usual care does not include any followup (which is often the case clinically) versus scheduled ongoing follow-up sessions, the potential for maintenance or improvement in CPAP use might be expected for the intervention group. Only studies with longer-term follow-up periods, on the order of 1-2 years, can begin to answer this question. 

This study was conducted at the Veterans Affairs Medical Center and was required to follow the policies and procedures in place at the time for the conduct of research studies. Some of the planned features for the website were deemed as possible privacy or security risks and were therefore not designed as part of the website, including email correspondence with providers, the use of forums or discussion boards for group members, the use of any open-ended questions or the use of an online diary, and website use tracking. Future iterations of an interactive website may benefit from including any one of these or other similar components. Clearly, lessons we learned from a previous study on group self-management intervention for OSA patients was that peer support was an important part of the management process [28]. Kate Lorig and colleagues have long espoused the importance of peer support [42]. 

When those participants in the MyCPAP group were asked about their use of the Internet and website, results showed that they significantly increased their use of the Internet to obtain information about sleep apnea from baseline to 4 months. But while they may have used the internet more to obtain information about sleep apnea, they did not increase their use of the internet to find information about their general health, nor did use of the MyCPAP website increase their confidence about the accuracy of health information found on the internet. In large part, this would appear to make sense because the MyCPAP website was known to them, as was the researcher team who developed it. In fact, we would have been surprised if knowledge and use of one disease-specific website generalized to others on the internet. This likely speaks to the discretion of those enrolled in our research study. 

We had an opportunity to take a closer look at the tracking of sleep apnea symptoms by those enrolled in the MyCPAP group. What seems to be apparent from Figures 5(a)–5(c) is that there is not a clear resolution of OSA-related symptoms even for these very good users of CPAP (i.e., use of CPAP more than 4 hrs/nt), if clear resolution is defined as a symptom rating of 2 or less (i.e., mild or no problem). This is consistent with one interesting unpublished result from one of our previous studies, which was that 60% of the sample still had self-reported disturbed sleep, as measured by the Pittsburgh Sleep Quality Inventory, even while being “compliant” with CPAP therapy at the 2-month time point. And Wickwire and colleagues found that those OSA patients with the complaint of insomnia had lower rates of CPAP use [43]. While placebo controlled trials of CPAP indicate that CPAP is highly efficacious at controlling apneas and hypopneas during sleep [44], it is becoming increasingly clear that this does not necessarily translate into the reduction of OSA symptoms to normal levels. Future studies will need to examine this issue in more depth, especially using new remote data capture methods that allow for more frequent assessments of OSA symptoms when using CPAP. 

Our group has examined the quality of sleep apnea information on the internet and found that only 2% of sleep apnea-related websites contained any graphical interactivity [45]. Since that time, other more general disease-oriented sites such as curetogether.com or patientslikeme.com have included the ability to track OSA-related symptoms to users who sign up for those kinds of services. 

The results of this paper found that while the confidence of OSA patients in the accuracy of the MyCPAP website increased over time, this did not generalize to other health-related websites. Our group also reviewed OSA-specific websites for measures of credibility (such as web certification, references, or display of authorship [46]), finding that fewer than 20% of the reviewed OSA websites included these kinds of measures of website credibility [45]. This makes sense given that the OSA participants in the current study knew the developers and the source of the MyCPAP website, while they may not know the source of the information in other health-related websites. And given that our review of OSA websites found that 80% did not include basic measures of website credibility, it is understandable that our OSA patients are leary of the information they found on the internet. Indeed, results of the 10th annual Health on the Net (HON) survey on health and medical information on the internet found that trustworthiness/credibility (96%) and accuracy of information (95%) to be the two most important factors related to the use of medical/health information on the internet [32]. When verifying whether the medical/health website is credible or not, users look to the source of the information (88%), the motivation of the owners of the website (68%), the URL (i.e., whether it is a commercial website or not; 66%), and the source of the funding for the website (55%). The reliability and quality of health information online continues to be a source of debate [47]. 

In terms of new ways to evaluate the credibility and accuracy of health and medical information on the internet, especially in this new era of interactive technologies, a recently published study provides such a framework [48]. Traditionally, these kinds of evaluations have included elements such as content credibility, interface usability, and overall appeal of website design. However, given the new “web 2.0” technologies that allow for more collaborative, adaptive and interactive components, the need for more “dynamic” evaluative criteria are important to account for the more interactive nature of these newer technologies. In particular, user-generated data, whether from online questionnaire tracking (e.g., Figures 5(a)–5(c) in this study) physiological measurement (e.g., blood pressure, weight), or from medical devices (e.g., CPAP or glucometer), is rapidly increasing. Also, the social nature of these websites is a rapidly expanding component, whether in terms of allowing peers to connect with each other, or allowing patients to connect with providers. The new framework includes formative, summative and outcome evaluation measures for five themes: people, content, technology, computer-mediated interaction, and health systems integration. While the utility of this framework will only be known over time, such efforts are needed to improve the design, development and efficiency of health-information websites. Ultimately, the goal is to improve the ability to provide accurate, reliable information and interactive applications for patients so that their health can be improved. 

The present study shows that the use of an interactive sleep apnea website can improve CPAP adherence, but that future iterations of such an intervention are needed to effect change in OSA outcomes. The use of such an intervention has the potential to be an important supplement to usual clinical care because of the power to provide educational and clinical information to the patient that is typically not provided to them. 

## Figures and Tables

**Table 1:** Descriptions of the components of the MyCPAP patient website.

Component	Description
The learning center	Basic education to inform patients about sleep apnea, CPAP, and collaborative management.
My CPAP data	Easy-to-read charts that show CPAP adherence (in hrs/nt) and CPAP efficacy data (disease severity as measured by number of apneas and hypopneas per hour) and amount of air leak (in liters/min).
My graphs	This section included both easy-to-complete individual items for patients to track, including sleepiness levels and other patient-selected OSA-related symptoms.
Troubleshooting guide	Interactive guide that allows patient to select the CPAP problem they are having; possible causes are discussed and solutions are listed.
CPAP user's manual	This component created animations for how to use the CPAP machine and it's associated features; how to clean mask and hose; how to use the humidifier.

**Table 2:** Baseline characteristics (Mean ± SD).

	Both groups (*N* = 241)	MyCPAP (*N* = 126)	Usual care (*N* = 114)	*P* value
Age	52.1 ± 13.3	52.7 ± 13.4	51.5 ± 13.2	0.52
Body mass index (BMI)	32.5 ± 8.0	32.6 ± 8.1	32.4 ± 8.1	0.86
Apnea-hypopnea index (AHI)	36.5 ± 25.9	36.3 ± 25.0	36.7 ± 27.3	0.91
Epworth sleepiness scale (ESS)	10.6 ± 5.3	10.7 ± 5.2	10.5 ± 5.4	0.75

**Table 3:** 

Measure	2 month visit				4 month visit			
	Both groups	UC (*N* = 114)	PC3 (*N* = 126)	*P* value	Both groups	UC (*N* = 114)	PC3 (*N* = 126)	*P* value
Epworth sleepiness scale	8.5 ± 5.4	8.1 ± 5.5	8.9 ± 5.3	NS	6.5 ± 4.2	5.7 ± 3.6	7.1 ± 4.5	NS
Sleep apnea quality of life	4.7 ± 2.1	4.5 ± 2.3	4.9 ± 1.9	NS	4.8 ± 2.3	4.6 ± 2.6	5.1 ± 2.0	NS
CES-D	8.5 ± 5.4	8.1 ± 5.5	8.9 ± 5.3	NS	7.9 ± 5.2	7.1 ± 4.9	8.6 ± 5.5	NS
Patient satisfaction	1.7 ± 1.2	1.8 ± 1.3	1.7 ± 1.1	NS	1.8 ± 1.2	1.9 ± 1.3	1.7 ± 1.1	NS
